# Anti-Inflammatory and Anti-Oxidative Effects of Myrtenol in the Rats with Allergic Asthma

**DOI:** 10.22037/ijpr.2019.1100749

**Published:** 2019

**Authors:** Mohammad Amin Rajizadeh, Hamid Najafipour, Mitra Samareh Fekr, Farzaneh Rostamzadeh, Elham Jafari, Mohammad Abbas Bejeshk, Yaser Masoumi-Ardakani

**Affiliations:** a *Physiology Research Center, Institute of Basic and Clinical Physiology Sciences, Kerman University of Medical Sciences, Kerman, Iran.*; b *Cardiovascular Research Center, Institute of Basic and Clinical Physiology Sciences and Department of Physiology, Afzalipour Medical Faculty, Kerman University of Medical Sciences, Kerman, Iran.*; c *Cardiovascular Research Center, Institute of Basic and Clinical Physiology Sciences, Kerman University of Medical Sciences, Kerman, Iran.*; d *Ph.D of Physiology, Endocrinology and Metabolism Research Center, Institute of Basic and Clinical Physiology Sciences, Kerman University of Medical Sciences, Kerman, Iran.*; e *Department of Pathology and Pathology and Stem Cells Research Center, Kerman University of Medical Science, Kerman, Iran.*; f *Gastroenterology and Hepatology Research Center, Institute of Basic and Clinical Physiology Sciences, Kerman University of Medical Sciences, Kerman, Iran. *; g *Physiology Research Center, Institute of Basic and Clinical Physiology Sciences, Kerman University of Medical Sciences, Kerman, Iran.*

**Keywords:** Allergic asthma, Myrtenol, Malondialdehyde, Superoxide dismutase, Glutathione peroxidase, Cytokines

## Abstract

The aim of the present study was to investigate the effect of Myrtenol, the active ingredient of Myrtle on the oxidant and anti-oxidant indices and cytokines in the allergic asthma. Allergic asthma was induced by ovalbumin (OVA) sensitization and inhalation in four groups of rats; Control, Asthma, Asthma + Dexamethasone and Asthma + Myrtenol. Myrtenol (50mg/kg) or Dexamethasone (2.5mg/kg) was administered intraperitoneally for 7 consecutive days after OVA inhalation. At the end, histopathological parameters, and interleukins (Interleukin-10 (IL10), Interferon gamma (IFN-γ) , interleukin-1β (IL-1β), Tumor Necrosis Factor α (TNF-α)), and oxidative stress biomarkers, Malondialdehyde (MDA), superoxide dismutase (SOD) and glutathione peroxidase (GPX) in the lung and serum were measured by hematoxylin and eosin staining and ELISA method, respectively. Myrtenol reduced the pathological changes in the lungs and airway endothelium (*P* < 0.01), (*P *< 0.5). The level of IL-1β (*P *< 0.05) and MDA in the serum and lung tissue (*P *< 0.01), (*P *< 0.05), and also the level of TNF-α (*P *< 0.05) in the lung tissue decreased in the Myrtenol group compared to the asthma group. Myrtenol increased the level of IL-10 (*P *< 0.05) and the activity of GPX in the lung tissue and serum (*P *< 0.001). Myrtenol may improve asthma by increasing the ratio of antioxidants to oxidants and reducing the ratio of pro-inflammatory to anti-inflammatory interleukins in the lung. Myrtenol is presented as a potent herbal medicine ingredient for the treatment of asthma.

## Introduction

Asthma is a heterogeneous disease, usually characterized by the airway inflammation. In developed countries, 10% of the adult population suffer from this disease, and its prevalence in children is even higher. In developing countries, the prevalence is lower but rising rapidly (1). The disease is characterized by a history of respiratory symptoms including wheezing, breathlessness, chest tightness, and coughing, varying in time and severity. Patients with periodic asthma experience airway obstruction and airway hyper-responsiveness (AHR) to various factors such as environmental allergens, airways infections, cold weather, exercise, and some medications (2).

In normal physiological conditions, reactive oxygen species (ROS) are continuously produced in the mitochondria, as a part of the normal cell metabolism (3), but enzymatic and non-enzymatic antioxidants protect blood and lung against adverse effects of oxidants. Asthma symptoms appear when increased oxidants overcome the antioxidant defense system (4). The increase in ROS in the lungs of asthmatic patients affects the barrier function of the alveolar epithelium and the capillaries endothelium, which results in the ROS spill over into the circulatory system (5). Oxidative changes lead to goblet cells hyperplasia, airway smooth muscle hypertrophy, subepithelial fibrosis, and eventually lead to the airway inflammation and remodeling (6).

Cytokines via mobilization, activating and survival of inflammatory cells intervene chronic inflammatory diseases, such as asthma (7). Pro-inflammatory cytokines such as tumor necrosis factor α (TNF-α) and interleukin-1β (IL-1β) play a definitive role in organizing the airway inflammatory responses (8). The number of macrophages expressing IL-1β and the expression of IL-1β in the airway epithelium of the asthmatic patients is increased (9). *In-vivo* and *in-vitro* studies on animal models have shown that after exposure to allergens, IL-1β stimulates Th2 production, activates eosinophils in the site of inflammation in the airway and increases release of cytokines such as interleukin-5 (IL-5). IL-5 stimulates airway inflammation, bronchial obstruction and increase AHR (10, 11). TNF-α is also a pro-inflammatory cytokine that is abundant in the airway of asthmatic patients (12). TNF-α acts as a chemo-attractant for neutrophils and eosinophils. It increases cytotoxic effects of eosinophils on the endothelial cells, activates T cells and increases the expression of epithelial cell adhesion molecules (13).

Interleukin-10 (IL10) and interferon gamma (IFN-γ) are protective cytokines against asthma (14). Interestingly, IL-10 has beneficial effects in controlling the airway remodeling. IL-10 also reduces type 1 collagen synthesis and smooth muscle cell proliferation (15). IFN-γ is a cytokine produced by Th2 and Th1 cells, which its level is reduced in the asthmatic patients (16). IFN-γ increases the expression of Th1 cytokines and suppresses Th2 cytokines (17).

Corticosteroids are a basic treatment for asthma, and most symptoms of this disease are controlled with the use of them alone or in combination with long-acting β-adrenergic agonists (18). Due to the chronic nature of asthma and the side effects of long-term use of corticosteroids it is necessary to find alternative medicines with fewer side effects**. **On the other hand, 5 to 10 percent of the asthmatic patients are strongly resistant to the treatment and they need new treatments (19). Therefore, the use of herbal medicines as an adjunct therapy to the standard treatments, in many chronic conditions such as asthma and lung fibrosis, is increasing. In our recent studies, the extract obtained from seeds of the Nigella sativa plant, Myrtel, and fennel had a beneficial effect on the lung fibrosis (20-22).

Myrtle is a medicinal plant known worldwide as a traditional medicine for the treatment of diseases (23). Myrtenol is one of the most effective ingredients of myrtle that contribute to the most important therapeutic effects of the plant (24). 

Considering the role of oxidants and cytokines in the development of asthma and airway inflammation and the anti-oxidative and anti-inflammatory effects of MYR, the present study aimed to investigate the effect of this herbal medicinal plant ingredient, in comparison with dexamethasone, on the levels of oxidants, antioxidants and several pro-inflammatory and anti-inflammatory cytokines in the lungs of rats with experimental allergic asthma. As increasing evidences indicates that spillover of inflammatory mediators into the circulation in inflammatory pulmonary diseases cause some degrees of systemic inflammation (25), we also assessed the level of these cytokines in the serum of the studied groups. 

## Experimental

*Materials*

In this study, 28 male Wistar rats weighing 200-250 gr, (aged 8-10 weeks) were purchased from Physiology Research Center of Kerman University of Medical Sciences and kept under controlled conditions (12 h light/12 h dark) with free access to water and normal rodent chow. The general protocols for animal care and use were approved by the Ethics Committee of Kerman University of Medical Sciences (Permission No. IR.KMU. REC.1395.660). 

The animals were randomly divided into 4 groups: Control/vehicle, asthma, asthma + dexamethasone (Dexa), and asthma + myrtenol (MYR). 

MYR and ovalbumin (OVA) were purchased from Sigma Aldrich (Gillingham, UK). Dexa was purchased from Iran Hormone (Iran). SOD and MAD assay kits were obtained from Nalondi (Iran). Glutathione peroxidase (GPX) activity colorimetric assay kit was purchased from Biovision (USA). Rat IL-10, IL-1β and TNF-α ELISA kits were obtained from Eastbiopharm (Hangzhou China).

*Induction of asthma and treatment protocols*

For induction of asthma, the animals were sensitized by intraperitoneal (IP) injection of 1 mg OVA and 200 µg Al (OH) 3 in 0.5 mL phosphate buffered saline (PBS) on days 0 and 7 (26). From days 15 to 42, the animals were exposed to aerosolized OVA (1%) in a closed chamber (40×40×70 cm) using a nebulizer (Omron CX3, Japan) for 30 min, every other day. The rats in the control/vehicle group received IP injection of PBS with aluminum hydroxide on days 0 and 7 and aerosolized with PBS for 30 min every other day from days 15 to 42 (26-28).

At the end of inhalational exposure, the rats in the asthma + MYR group received 50 mg/kg MYR (24) and in the asthma + Dexa group they received 2.5 mg/kg Dexa (29), both daily and intraperitonealy, for one week (30).

*Preparation of lung tissue sections and serum*

On day 50, the rats were euthanized under deep anesthesia by ketamine (80 mg/Kg) and xylazine (10 mg/Kg) injection, and their blood was collected and sera were separated by centrifugation and stored at -80 °C. The middle lobe of right lung was removed and frozen in liquid nitrogen and stored at −80 °C. The prepared tissues and sera were used for the measurement of oxidants, antioxidants, and interleukins. 

Left lungs of the rats were removed and placed into 10% buffered formalin. Then the tissues were paraffin blocked, and 4-μm slices were obtained and stained with Hematoxylin/Eosin (H&E). The sections were examined microscopically by two pathologists who were blind to the animal groups. The pathologic indices included pneumocyte and fibroblastic hypertrophy and hyperplasia, edematous and degenerative changes, necrosis and airway epithelial denudation, atelectasis, hyperemia, hemorrhage and exudation. The indices were scored using a 5-scale grading system: 0 = no lesion, 1 = slight, 2 = mild, 3 = moderate and 4 = severe lesions (31).

The damage to the epithelium was evaluated with a grading lesion score as fallow: 1 = normal appearance, 2 = observable loss of cilia, or degeneration or necrosis of less than one-fourth of the ciliated cells without epithelial sloughing, 3 = observable epithelial sloughing and degeneration or necrosis of less than one-fourth of the ciliated cells, 4 = epithelial sloughing and degeneration or necrosis between one-fourth and one-half of the ciliated cells, and 5 = epithelial sloughing and degeneration or necrosis more than one-half of the ciliated cell (32).

*Measurement of oxidants, antioxidants and interleukins*

Superoxide dismutase (SOD) was measured using a colorimetric assay based on the ability of SOD to inhibit the auto-oxidation of pyrogallol. Briefly, 50 mg of lung tissue was homogenized on ice in 250 μL of lysis buffer. The sample was then centrifuged at 12000 rpm for 5 min at 4 °C. 50 μL of the supernatant was used to measure the activity of SOD using the related kit, according to the manufacturer′s instructions. 

To measure GPX activity in the lung tissue, 100 mg of the tissue was homogenized on ice in 200 µL assay buffer. The homogenate was centrifuged at 10000 x g for 15 min at 4 °C and the supernatant was used. The serum was tested directly. GPX activity was evaluated by reducing Cumene Hydroperoxide and oxidizing glutathione (GSH) to oxidized glutathione (GSSG). The generated GSSG was reduced to GSH with the consumption of nicotinamide adenine dinucleotide phosphate hydrogen (NADPH) by Glutathione Reductase. The decreased NADPH (that is proportional to GPX activity) was measured at 340 nm.

Malondialdehyde (MDA) level, as an index of lipid peroxidation, was estimated by the concentration of thiobarbituric acid reactive substances (TBARS). 250 μL of the serum or homogenized tissue samples were used to measure it according to the kit′s instructions. Tissue samples were lysed using 1.5% potassium chloride solution. The homogenate was centrifuged at 1200 rpm for 10 min.

Quantitative assessments of IL-1β, TNF- α, IL-10 and INF-γ were conducted using double– antibody sandwich Enzyme- Linked Immunosorbent Assay (ELISA) kits based on their manufacturers’ instructions.

*Statistical analysis*

Quantitative data are presented as Mean ± SEM. After analyzing the normality of the data using Shapiro-Wilk statistical test and were analyzed using ANOVA followed by Tukey′s post-hoc test. Scored data (lung pathology indices) were analyzed using nonparametric tests (Kruskal Wallis test) with the Bonferroni correction. P values less than 0.05 were considered as significant.

## Results

*The effect of Mythonol on pathological indices*

**Figure 1:**
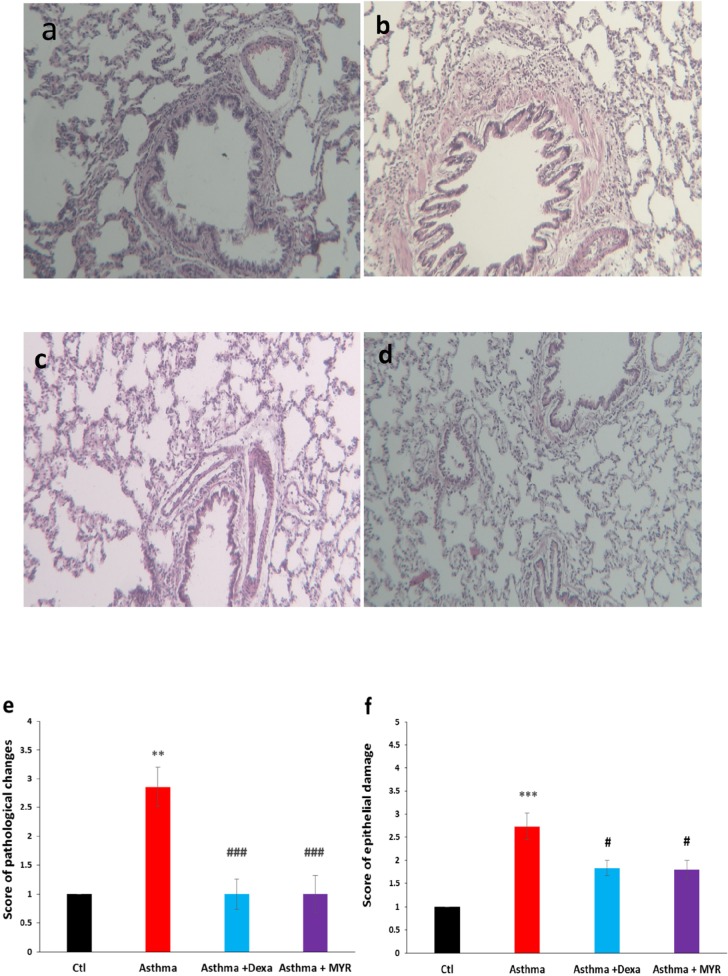
Micrographs of the lung stained with H & E showing pathologic changes in the bronchial epithelium, inflammatory cell infiltration, thickening of subepithelial smooth muscle layer, hyperemia and atelectasis in the asthma group. MYR, similar to Dexa, decreased inflammation and other abnormalities (400 x magnifications). a: Control, b: Asthma. c: Dexa, d: MYR. e: The score of pathologic changes in the lung and f: the score of epithelial damage of bronchi in the studied groups. Ctl: Control, Dexa: Dexametazone, MYR: Myrtenol. ** = *P *< 0.01 and ***= *P *< 0.001 vs Ctl, ### =*P *< 0.001 vs Asthma. The data were analyzed using Kruskal Wallis and Mann-Whitney tests

The score of pathological changes of the lung increased in the asthma group compared to the control group (*P *< 0.01). Treatment with Dexa and MYR significantly reduced the pathological changes (*P *< 0.001) (Figure 1e). The bronchial epithelium damages increased in the asthma group compared to the control group (*P *< 0.001) and Dexa and MYR reduced these damages (*P *< 0.05) (Figure 1f).

*The effect of Myrtenol on oxidative and anti-oxidative indices in the lung tissues and serum*

**Figure 2:**
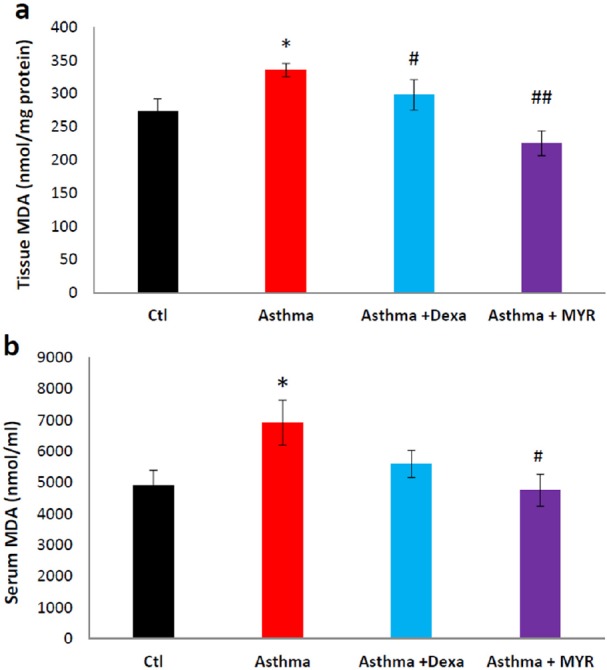
The effect of Myrtenol on the MAD level in lung tissue (a) and in serum (b) of asthmatic rats. *= *P *< 0.05 vs Ctl, # = *P *<0.05 and ## = *P *< 0.01 vs Asthma. The data were analyzed using ANOVA followed by Tukey′s post-hoc test

The level of MDA in the lung tissue increased in the asthma group (*P *< 0.05). Dexa and MYR reduced MDA levels compared to the asthma group (*P *< 0.05, *P *< 0.01) (Figure 2a). MDA also increased in the serum of asthmatic rats, and MYR decreased its level compared to the asthma group (*P *< 0.05) (Figure 2b).

**Figure 3:**
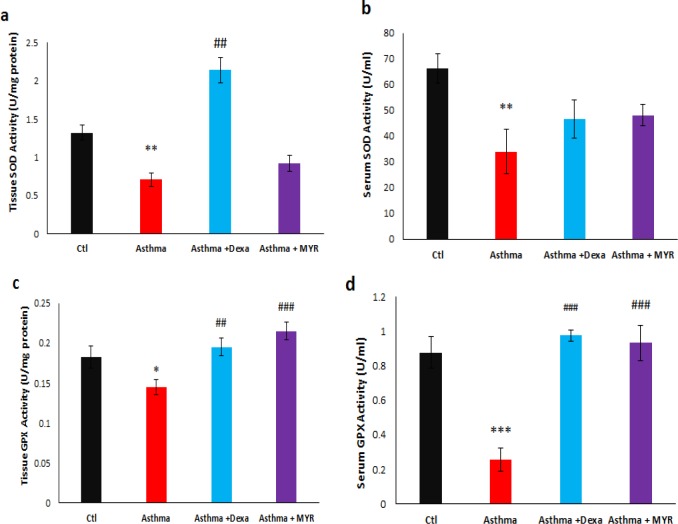
The effect of Myrtenol on the levels of SOD and GPX activity in the lung tissue (a, c) and serum (b, d) of asthmatic rats

The levels of superoxide dismutase (SOD) activity in the lung tissue decreased in the asthma group (*P *< 0.01). Dexa increased the levels of SOD activity compared to the asthma group (*P *< 0.01), but MYR could not affect its level (Figure 3a). Serum SOD activity also decreased in asthmatic rats (*P *< 0.01). Neither Dexa nor MYR had a significant effect on the level of SOD activity in the serum (Figure 3b).

The level of Glutathione peroxidase (GPX) activity decreased in the lung tissue (*P *< 0.05) and serum (*P *< 0.001) of asthmatic rats. Both Dexa and MYR increased the level of GPX activity in the lung tissue (*P *< 0.01, *P *< 0.001) (Figure 3c) and in serum (*P *< 0.001) (Figure 3c, d).

*The effect of Myrtenol on the inflammatory indices*

**Figure 4.**
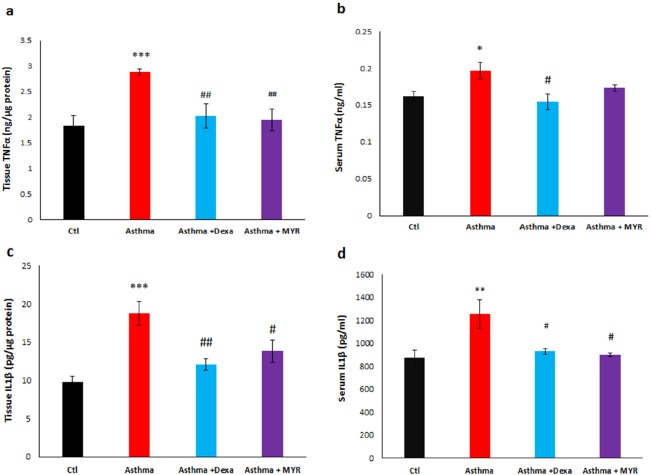
The effect of Myrtenol on the levels of TNF-α and IL-1β in the lung tissue (a, c) and serum (b, d) of asthmatic rats

The level of TNF-α increased in the lung tissue of the asthma group (*P *< 0.001). Dexa and MYR reduced the levels of TNF-α compared to the asthma group (*P *< 0.01) (Figure 4a). TNF-α also increased in the serum of the asthmatic rats (*P *< 0.05). Dexa reduced the level of serum TNF-α compared to the asthma group (*P *< 0.05) but MYR did not affect it (Figure 4b).

The level of IL-1β increased in the lung tissue (*P *< 0.001) and serum (*P *< 0.01) of the asthmatic rats. Both Dexa and MYR decreased the level of IL-1β in the lung tissue (*P *< 0.01, *P *< 0.05) (Figure 4c) and in serum (*P *< 0.05) (Figure 4d) compared to the asthma group.

**Figure 5:**
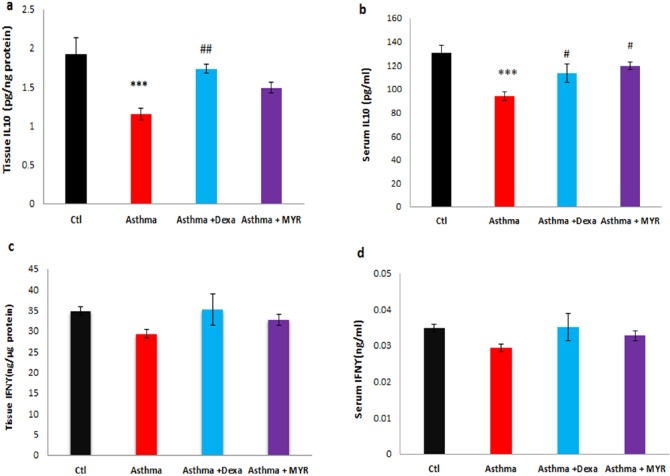
The effect of Myrtenol on the levels of IL-10 and IFN-γ in the lung tissue (a, c) and serum (b,d) of asthmatic rats. ***

The level of IL-10 decreased in the lung tissue of the asthma group (*P *< 0.001). Dexa increased the level of IL-10 compared to the asthma group (*P *< 0.01), but MYR could not increase its level (Figure 5a). IL-10 level decreased in the serum of asthmatic rats (*P *< 0.001). Dexa and MYR both increased the level of IL-10 compared to the asthma group (*P *< 0.05) (Figure 5b).

The levels of INF-γ in the lung tissue and serum among the groups were not significantly different (Figure 5c, d).

## Discussion

The findings of this study showed that Myrtenol, the active ingredient of Myrtle, reduces the pathological changes in the lung and bronchial epithelium of the experimental asthmatic animals. Also, it reduced the level of inflammatory cytokines TNF-α and IL-1β and increased the level of anti-inflammatory cytokine IL-10.

Many studies have demonstrated that the level of oxidants in the lung increases in asthma (6,33, 34). Oxidative stress is not limited to the lung, but reactive oxygen species are also likely to be flowed from the lung tissue into the bloodstream in the asthmatic patients. Therefore, in addition to the importance of oxidative stress in the induction of inflammation in the lungs, it causes secondary inflammation to be spread via bloodstream throughout the body. Systemic and lung inflammation in the asthma may result in cardiovascular complications (25). It has been revealed that there is a direct relationship between the oxidant-antioxidant imbalance in the airways and in the blood (33). The findings of this study also showed that the level of MDA increased in the lung and serum of asthmatic rats, and MYR inhibited this increase. Compared to Dexa, MYR had more prominent effect on the level of MDA in the lung and serum (Figure 2). Therefore, MYR in addition to its beneficial effect in the treatment of asthma may reduce cardiovascular complications in asthma. It has also revealed that MYR reduces the myocardial ischemia-reperfusion injury by reducing the oxidative stress (35). 

* =*P *< 0.05, ** =*P *< 0.01 and *** = *P*< 0.001 vs Ctl, ## = *P *< 0.01 and ### = *P *< 0.001, vs Asthma. The data were analyzed using

ANOVA followed by Tukey′s post-hoc test.

* =*P *< 0.05, ** =*P *< 0.01 and ***= *P *< 0.001 vs Ctl, # = *P *< 0.05, ## = *P *< 0.01 and ### = *P *< 0.001 vs Asthma. The data were analyzed using ANOVA followed by Tukey′s post-hoc test.

=*P*<0.001 vs Ctl, # = *P *< 0.05, ## = *P *< 0.01 vs Asthma. The data were analyzed using ANOVA followed by Tukey′s post-hoc test.

There is no evidence of toxicity in oral administration of MYR in animals. One study has reported the lethal dose of 296 mg/kg by IP administration (24). The anti-oxidative effect of the dose administered in this study (50 mg/kg), has been shown in the other studies (24, 35).

GPX and SOD are the main antioxidants in the lungs, and their activity is reduced in asthma (36). The results of this study indicated that MYR increases the levels of GPX activity in the lung and serum of asthmatic rats. The reduction in GPX levels inhibits the production of Th1-dependent cytokines and increases the Th2-related responses (37). Therefore, MYR can reduce airway inflammation and improve symptoms of asthma by increasing the GPX levels in the lung, establishing the oxidants-antioxidants balance, and regulating the production of inflammatory and anti-inflammatory cytokines.

In the asthmatic rats, TNF-α level increased in the serum and in the lung, and MYR could reduce its tissue level efficiently (Figure 4) (16). TNF-α is a pro-inflammatory cytokine, which has been implicated in many aspects of the airway pathology in asthma. Preliminary studies have demonstrated that lung function, quality of life, and hyper-responsiveness improved by the use of anti-TNF-α drugs (38).

IL-1β is the main inflammatory mediator in asthma. By activating NF-Kβ, IL-1β increases the expression of cytokines, chemokines, complement system proteins, and immune receptors that contribute to the inflammation, and also increases the responsiveness of the bronchial smooth muscle (39). Therefore, IL-1β is a potential therapeutic target in asthma. Figure 5 shows the increase of IL-1β levels in the serum and lung of asthmatic rats and that MYR decreased its level in both. As a result, MYR with the reduction of IL-1β, the key interleukin, can be effective in the treatment of asthma. 

IL-10 is a regulatory cytokine that is proposed for the treatment of asthma due to its anti-inflammatory properties (40). The results of this study indicated that MYR can also increase the tissue and serum levels of IL-10, which is the strongest anti-inflammatory cytokine.

The results of the present study showed no significant changes in the level of IFN-γ in the lung tissue or sera of the studied groups. The experimental and clinical evidences indicated that changes in IFN-γ level is related to the severity and duration of asthma (16, 41). It has been shown that the number of IFN-γ-producing CD4+ T increased in peribronchial lymph nodes after chronic allergen challenge but not in acute challenge (16). Probably the cause of not changing in the level of IFN-γ in the present study is that our model of asthma is probably not sufficiently chronic.

The results of this study also confirm the findings of the previous studies (25, 42) that probably occurred in allergic asthma spillover of inflammatory mediators into the circulation. Adipose tissue-mediated inflammation has been discussed as a connecting link of systemic inflammation in inflammatory pulmonary diseases (25).

Overall MYR can modulate oxidant/antioxidant and pro-inflammatory/anti-inflammatory balance in the lung tissue and serum. It has been revealed that airway inflammation is associated with increased systemic inflammation in asthma (42). Therefore, it seems that MYR can be considered as a therapeutic target in the treatment of asthma in both systemic and inhalation forms. It seems that the drugs that affect the synthesis of several cytokines probably have a better effect than the specific cytokine inhibitors (1). Consequently, MYR may be found more useful in the treatment of asthma.

## Conclusion

MYR can reduce inflammation and tissue damages in the asthma, by reducing the pro-inflammatory cytokines and increasing the anti-inflammatory cytokines. It can also improve the antioxidant-oxidant balance in the lungs of asthmatic rats. Therefore, MYR can be used to treat asthma without suppressing the immune system. However, clinical trials are needed to be conducted to confirm its effects at the clinical level.
